# Randomized Phase II trial of paclitaxel and carboplatin followed by gemcitabine switch-maintenance therapy versus gemcitabine and carboplatin followed by gemcitabine continuation-maintenance therapy in previously untreated advanced non-small cell lung cancer

**DOI:** 10.1186/1756-0500-6-3

**Published:** 2013-01-03

**Authors:** Seigo Minami, Takashi Kijima, Takayuki Shiroyama, Kohei Okafuji, Tomonori Hirashima, Junji Uchida, Fumio Imamura, Tadashi Osaki, Takeshi Nakatani, Yoshitaka Ogata, Suguru Yamamoto, Yoshinobu Namba, Tomoyuki Otsuka, Isao Tachibana, Kiyoshi Komuta, Ichiro Kawase

**Affiliations:** 1 Department of Respiratory Medicine, Osaka Police Hospital, Osaka, Japan; 2 Department of Respiratory Medicine, Allergy and Rheumatic Diseases, Osaka University Graduate School of Medicine, Osaka, Japan; 3 Department of Thoracic Malignancy, Osaka Prefectural Medical Center for Respiratory and Allergic Diseases, Osaka, Japan; 4 Department of Pulmonary Oncology, Osaka Medical Center for Cancer and Cardiovascular Diseases, Osaka, Japan; 5 Department of Respiratory Medicine, Kinki Central Hospital for Mutual Aid Association of Public School Teachers, Hyogo, Japan; 6 Department of Respiratory Medicine, National Hospital Organization Toneyama National Hospital, Osaka, Japan; 7 Department of Internal Medicine, Nishinomiya Municipal Central Hospital, Hyogo, Japan

**Keywords:** Carboplatin, Gemcitabine, Paclitaxel, Switch-maintenance, Continuation-maintenance, Non-small cell lung cancer (NSCLC), Randomized phase II

## Abstract

**Background:**

In recent years, maintenance chemotherapy is increasingly being recognized as a new treatment strategy to improve the outcome of advanced non-small cell lung cancer (NSCLC). However, the optimal maintenance strategy is still controversial. Gemcitabine is a promising candidate for single-agent maintenance therapy because of little toxicity and good tolerability. We have conducted a randomized phase II study to evaluate the validity of single-agent maintenance chemotherapy of gemcitabine and to compare continuation- and switch-maintenance.

**Methods:**

Chemonaïve patients with stage IIIB/IV NSCLC were randomly assigned 1:1 to either arm A or B. Patients received paclitaxel (200 mg/m^2^, day 1) plus carboplatin (AUC 6 mg/mL/min, day 1) every 3 weeks in arm A, or gemcitabine (1000 mg/m^2^, days 1 and 8) plus carboplatin (AUC 5 mg/mL/min, day1) every 3 weeks in arm B. Non-progressive patients following 3 cycles of induction chemotherapy received maintenance gemcitabine (1000 mg/m^2^, days 1 and 8) every 3 weeks. (Trial registration: UMIN000008252)

**Results:**

The study was stopped because of delayed accrual at interim analysis. Of the randomly assigned 50 patients, 49 except for one in arm B were evaluable. Median progression-free survival (PFS) was 4.6 months for arm A *vs*. 3.5 months for arm B (HR = 1.03; 95% CI, 0.45–2.27; *p* = 0.95) and median overall survival (OS) was 15.0 months for arm A *vs*. 14.8 months for arm B (HR = 0.79; 95% CI, 0.40–1.51; *p* = 0.60), showing no difference between the two arms. The response rate, disease control rate, and the transit rate to maintenance phase were 36.0% (9/25), 64.0% (16/25), and 48% (12/25) for arm A *vs*. 16.7% (4/24), 50.0% (12/24), and 33% (8/24) for arm B, which were also statistically similar between the two arms (*p* = 0.13, *p* = 0.32, and *p* = 0.30, respectively). Both induction regimens were tolerable, except that more patients experienced peripheral neuropathy in arm A. Toxicities during the maintenance phase were also minimal.

**Conclusion:**

Survival and overall response were not significantly different between the two arms. Gemcitabine may be well-tolerable and feasible for maintenance therapy.

## Background

Lung cancer is the leading cause of cancer death worldwide [1]. Non-small cell lung cancer (NSCLC) accounts for 85% of all lung cancer cases and most of them are found to have locally advanced or metastatic diseases at the time of diagnosis. However, the efficacy of standard platinum-based regimens in the first-line setting for advanced NSCLC has reached a plateau. Recently, maintenance therapy is increasingly being approved as a new treatment paradigm to improve the outcome for advanced NSCLC. New generation cytotoxic agents such as paclitaxel [2], vinorelbine [3], docetaxel [4], gemcitabine [5,6], and pemetrexed [7], and molecular-targeted agents such as gefitinib [8], erlotinib [9] and bevacizumab [10,11], have been evaluated for their efficacy as single-agent maintenance therapy. Among these wide range of agents, gemcitabine (Gemzar®, Eli Lilly & Co., Indianapolis, IN, USA) is a pyrimidine antimetabolite that has demonstrated antitumor activities in diverse tumor types. It is not only one of the standard drugs in the regimens for untreated advanced-stage NSCLC [12-15], but also active against recurrent NSCLC after platinum-based chemotherapy [16-18]. In addition to these antitumor activities, it is also one of the promising candidates for maintenance use because of its low cumulative toxicities [5,6].

Maintenance chemotherapy can be broadly categorized as switch-maintenance and continuation-maintenance. The former is defined as maintenance treatment with different drugs from those used in the induction regimen, while the latter is with one or two of the drugs used in the induction regimen. In the latest meta-analysis of maintenance chemotherapy in advanced NSCLC [19], the differences in overall survival (OS) and progression-free survival (PFS) between the two maintenance strategies were not statistically significant. However, no study has compared these two maintenance strategies directly. Therefore, we conducted an investigational randomized phase II study to evaluate the validity of gemcitabine for single-agent maintenance chemotherapy and to compare switch- and continuation-maintenance.

## Method

### Objectives and study design

This trial was a randomized, open-label, multi-centered, phase II study. The primary objective was to investigate PFS. Secondary objectives were to investigate the objective response rate (RR) and safety. The study protocol was approved by each institutional ethics committee and adhered to the principles outlined in the Guideline for Good Clinical Practice (January 1997) and Declaration of Helsinki (1996). Written informed consent was obtained from all patients before commencement of the study.

### Patient selection

Patients were enrolled when they met all the following entry criteria: (1) histologically or cytologically confirmed NSCLC with chemotherapy-naïve stage IIIB/IV; (2) 20 ≤ age < 75 years; (3) having measurable disease according to Response Evaluation Criteria in Solid Tumors (RECIST) version 1.0; (4) an Eastern Cooperative Oncology Group (ECOG) performance status (PS) grade of 0–1; (5) adequate hematologic (absolute white blood cell count ≥ 4000/μL, neutrophil count ≥ 1500/μL, platelets ≥ 100,000/μL, and hemoglobin ≥ 10.0 g/dL), renal (serum creatinine ≤ 1.2 mg/dL and creatinin clearance calculated by Cockcroft-Gault formula ≥ 60 mL/min), liver (serum total bilirubin ≤ 1.5 mg/dL, aspartate aminotransferase and alanine aminotransferase ≤ 100 IU/L), and respiratory (SpO_2_ ≥ 95% under room air) functions; (6) estimated life expectancy of more than 3 months; (7) written informed consent. Patients with asymptomatic brain metastases were also eligible. On the other hand, exclusion criteria were; (1) clinically significant complications or unstable medical conditions; (2) recurrence after resection, and curative or palliative thoracic radiotherapy for primary tumor irrespective of irradiation dose; (3) palliative extra-thoracic radiotherapy within 2 weeks; (4) pregnancy, lactation, suspicion of being pregnant; (5) other neoplasm with progression-free time under 5 years.

### Treatment plan

Patients were randomly assigned to arm A or B using dynamic allocation by institution, gender, and PS. In arm A, carboplatin (AUC 6 mg/mL/min, day 1) and paclitaxel (200 mg/m^2^, day 1) were administered intravenously (i.v.) every 3 weeks. In arm B, carboplatin (AUC 5 mg/mL/min, day 1) and gemcitabine (1000 mg/m^2^, days 1 and 8) were i.v. administered every 3 weeks. The number of cycles for induction chemotherapy was defined 3 by the following two reasons; (1) no evidence for additional clinical benefit by continuing cisplatin-containing chemotherapy beyond 3 cycles was demonstrated [20], (2) the mean and median number of administered platinum doublet chemotherapy was only 3 in Japanese Four-Arm Cooperative Study (FACS) [13]. After 3 cycles, non-progressive patients underwent maintenance gemcitabine (1000 mg/m^2^, days 1 and 8) every 3 weeks. Treatment was continued until disease progression, unacceptable toxicity, or withdrawal of consent. The glomerular filtration rate and carboplatin dose were calculated using Cockcroft-Gault and Calvert formula, respectively. Serum creatinine concentrations of the enzyme method were calibrated to those of the noncompensated Jaffé’s method by adding 0.2 mg/dL. Unless all the starting criteria defined in the protocol were met, administration of drugs on days 1 and 8 was postponed. Discontinuation criteria of protocol treatment included; (1) delay of the start of the next course longer than one week, (2) delay of gemcitabine administration on day 8 longer than 2 weeks, (3) necessity of third-time dose reduction, (4) documented disease progression and (5) patient’s refusal to continue the protocol therapy.

### Assessments

Required baseline assessments included chest and abdominal computed tomography (CT), cranial CT or magnetic resonance imaging (MRI), and bone scintigraphy or positron emission tomography (PET) within 4 weeks before enrollment. Overall response was evaluated according to RECIST version 1.0 after the first and third cycles of induction chemotherapy, and then every 2 cycles during maintenance chemotherapy. Toxicity was graded by the National Cancer Institute-Common Terminology Criteria for Adverse Events (NCI-CTCAE) version 3.0.

### Statistical analyses

The sample size was based on the assumption that the hazard ratio (HR) of arm B to arm A for PFS would be approximately 1.5. To select a better treatment arm, with two-sided alpha of 5% and a power of 90%, at least 94 evaluable patients and 74 non-progressive responders were needed. Given the possibility of deviation from assessment, 100 patients (50 per arm) were necessary. The evaluable population for overall response included all patients, defined as those without major protocol violation, who had received at least one cycle chemotherapy and had at least two response assessment over 6 weeks after the enrollment unless determination of objective progressive disease (PD). Patients who received any protocol therapy without major protocol violation were considered evaluable for PFS, OS and safety. PFS and OS were evaluated by Kaplan-Meier method. Differences between the two arms were evaluated by Pearson’s chi-square test and the log-rank test, and the HR was calculated by Cox regression model.

## Results

Although enrollment to this study was planned to complete within 2 years, only half the required samples were accrued in the time-period. During the study period, pemetrexed and bevacizumab became commercially available in Japan in May 2009 and November 2009, respectively. Therefore, this study was terminated at that time because of delayed accrual and fear of biased histology in favor of squamous cell carcinoma.

### Patient demographics

From December 2007 to November 2009, a total of 50 patients, 25 randomly assigned to each arm, were enrolled from seven medical institutions. The baseline characteristics were similar between the two arms, except more male patients were enrolled in arm A (Table 1). Data on smoking habits and the epidermal growth factor receptor (EGFR) mutation status were not collected.

**Table 1:** Patients’ characteristics

	**arm A (n = 25)**	**arm B (n = 25)**	***p*-value**
Sex			0.11
Male	21	16	
Female	4	9	
Age			0.81
median	63	65	
range	55-74	45-74	
Histology			0.69
Adenocarcinoma	17	18	
Large cell carcinoma	0	1	
Squamous	6	5	
Others	2	1	
Stage			0.56
IIIB	10	8	
IV	15	17	
PS			0.54
0	9	6	
1	16	19	

### Treatment

One patient in arm B was excluded from analyses because of a major protocol violation during induction phase. Of the rest 49 evaluable patients, 68% (17/25) in arm A and 50% (12/24) in arm B completed 3 cycles of induction chemotherapy.

Eight patients in arm A dropped out during induction phase because of PD (n = 5), physician’s decision (n = 2), persistent anemia (n = 1), while 13 in arm B did because of PD (n = 3), hematologic AEs (n = 4), persistent ALT elevation (n = 2), physician’s decision (n = 1), and complicated disease (n = 3). Complicated diseases included sudden onset ovarian torsion that required emergent surgery, aspiration pneumonia and pyothorax that required hospitalization and antibiotics therapy. During transition from completion of induction phase to start of maintenance phase, 5 patients in arm A and 3 in arm B dropped out. In arm A, 3 patients resulted in PD, one kept SD but failed to recover anemia, and one achieved PR but suffered from bacterial pneumonia at the end of induction phase. In arm B, 2 patients resulted in PD at the end of induction phase despite having kept SD during induction phase, and one kept SD after induction phase but rapidly progressed until commencement of maintenance phase.

**Figure 1:**
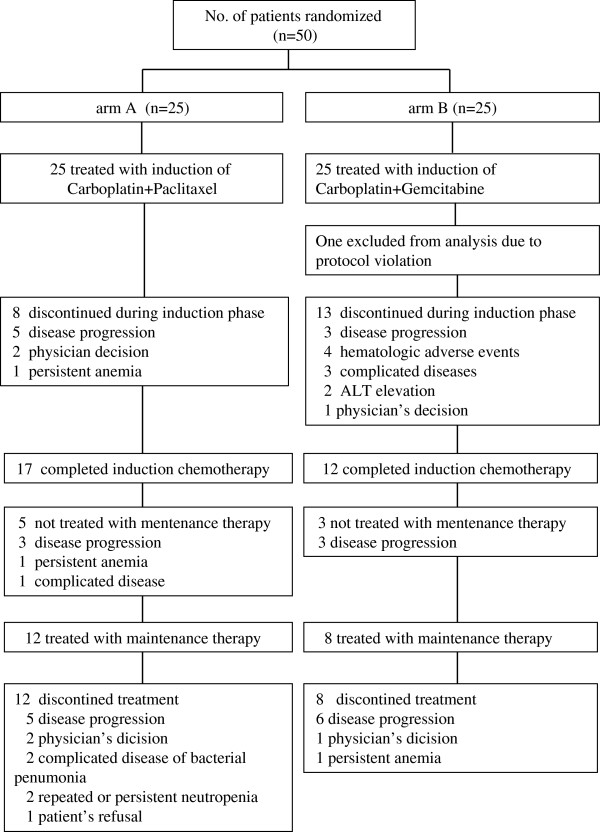
CONSORT flow chart for the study.

Finally, only 12 patients (48%) in arm A and 8 (33%) in arm B received gemcitabine maintenance therapy (*p* = 0.25) (Figure 1). In both arms, there was no statistically significant difference in baseline characteristics between discontinued patients during induction phase and those received gemcitabine maintenance therapy (data not shown). The average number of maintenance cycles delivered was 4.66 (range 1 to 11) in arm A and 6.75 (range 1 to 36) in arm B. All the 20 patients discontinued maintenance treatment as of November 2011 because of PD (n = 11), physician’s decision (n = 3), patient’s refusal (n = 2), and treatment-related AEs (n = 4; 2 repeated and/or persistent neutropenia, 1 persistent anemia and 1 bacterial pneumonia).

### Efficacy

The follow-up data were collected up to November 8th, 2011. The median follow-up time was 13.6 months (95% CI, 8.6 to 17.1 months). At the time of the data collection, 2 patients in arm A and 5 in arm B were still alive. There was no patient still on maintenance treatment and 2 in arm A and 2 in arm B were lost to follow-up.

### PFS and OS

**Figure 2:**
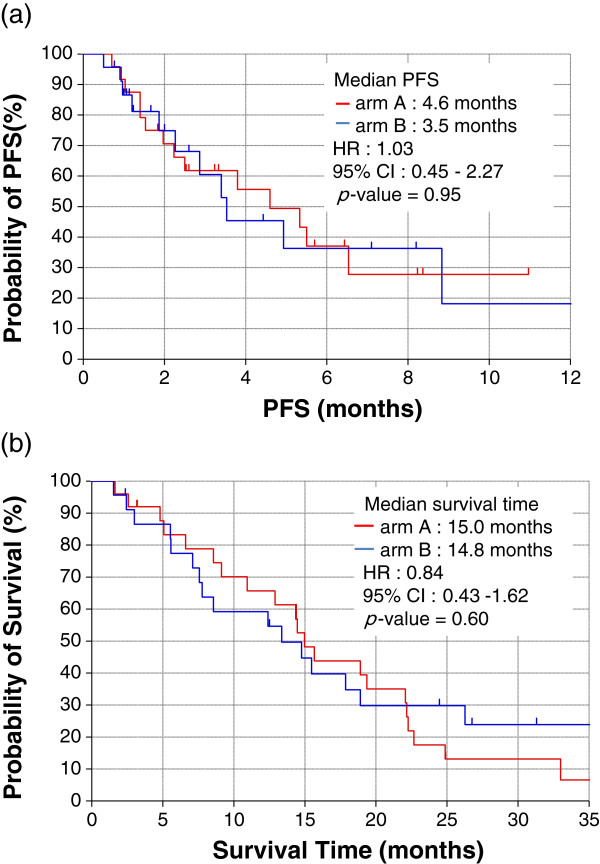
Kaplan-Meier curves of PFS(a) and OS(b).

The median PFS and OS were 4.6 months (95% CI, 2.0 to not available) and 15.0 months (95% CI, 9.1 to 22.1 months) in arm A, and 3.5 months (95% CI, 1.9 to not available) and 14.8 months (95% CI, 7.6 to 26.3 months) in arm B, respectively. There was no statistical difference between the two arms in terms of both PFS (*p* = 0.95) and OS (*p* = 0.60) (Figure 2).

### Overall response

In the induction therapy, 9 patients in arm A and 4 in arm B achieved partial response (PR), and 7 and 8 kept stable disease (SD), respectively. Consequently, the objective RR (36.0% *vs*. 16.7%, *p* = 0.13) and disease control rate (DCR) (64.0% *vs*. 50.0%, *p* = 0.32) showed no significant differences between the two arms (Table 2). Of 7 SD patients in arm A, one achieved PR after 3 cycles of maintenance therapy. In 11 patients with squamous cell carcinoma, one patient achieved PR, 2 kept SD and 3 progressed in arm A, while one patient kept SD, 2 progressed and 2 were not evaluated in arm B.

**Table 2:** Efficacy

	**arm A**	**arm B**	
	**(n = 25)**	**(n = 24)**	***p*-value**
CR	0	0	
PR	9	4	
SD	7	8	
PD	8	5	
NE	1	7	
RR(%)	36.0	16.7	0.13
DCR(%)	64.0	50.0	0.32

### Safety

The incidental rates of grade 3-4 AEs were low. During induction phase, hematologic AEs of grade 4 neutropenia (9 in arm A and 4 in arm B), grade 3 anemia (1 in arm B), and grade 4 thrombocytopenia (2 in arm B) were observed. Severe non-hematologic AEs included grade 3 skin rash (1 in each arm), grade 3 aminotransferase increase (1 in arm A and 3 in arm B), grade 3 febrile neutropenia (1 in arm A), and grade 3 peripheral sensory neuropathy (2 in arm A). Of all AEs, peripheral sensory neuropathy, arthralgia, and myalgia peculiar to paclitaxel were more frequently observed in arm A (*p* = 0.02, 0.04 and 0.02, respectively). Neutropenia also tended to be more frequent in arm A (*p* = 0.06) (Table 3). During maintenance phase, hematologic AEs of grade 3-4 neutropenia (3 in arm A and 2 in arm B), and grade 3 thrombocytopenia (1 in arm A) were observed. Non-hematologic AEs of grade 3-4 were not experienced (Table 4).

**Table 3:** Toxicities during induction chemotherapy

	**arm A (n = 25)**				**arm B (n = 24)**			
Grade	1	2	3	4	1	2	3	4
Leukopenia	1	2	1	-	-	2	2	1
Neutropenia	-	-	8	9	-	1	5	4
Neutropenic fever	-	-	1	-	-	-	-	-
Thrombocytopenia	-	-	-	-	1	4	1	2
Amenia	1	-	-	-	-	-	1	-
Low Hemoglobin	-	-	-	-	1	2	-	-
AST/ALT	3	2	1	-	-	2	3	-
Nausea	3	1	-	-	3	1	-	-
Pain	-	-	-	-	1	1	-	-
Arthralgia	2	2	-	-	-	-	-	-
Myalgia	2	3	-	-	-	-	-	-
Skin rash	1	2	1	-	1	2	1	-
Conspitation	2	-	-	-	3	-	-	-
Fever	2	-	-	-	-	2	-	-
Fatigue	3	1	-	-	1	1	-	-
Peripheral Neurotoxicies	2	1	2	-	-	-	-	-
Pruritus	-	1	-	-	-	1	-	-
Appetite loss	1	2	-	-	1	1	-	-

**Table 4:** All toxicities of grade 1-4 and severe toxicities of grade 3-4 during gemcitabine maintenance

	**ALL (n = 20)**		**arm A (n = 12)**		**arm B (n = 8)**	
	**G1-4**	**G3-4**	**G1-4**	**G3-4**	**G1-4**	**G3-4**
Leukopenia	1	-	1	-	-	-
Neutropenia	6	5	3	3	3	2
Thrombocytopenia	3	1	3	1	-	-
reduced Hb	1	-	-	-	1	-
Anemia	1	-	1	-	-	-
Peripheral neurotoxicities	1	-	1	-	-	-
Skin rash	1	-	1	-	-	-
Fever	1	-	1	-	-	-
Pain	1	-	-	-	1	-
Cutaneous injection reaction	1	-	-	-	1	-
Liver abnormality	2	-	2	-	-	-
Complicated pneumonia	1	-	1	-	-	-

### Post-protocol treatment

After the protocol therapy was discontinued, 21 patients (84%) in arm A and 18 (75%) in arm B received systemic chemotherapy. Among the 20 patients who received maintenance therapy, 10 (83.3%) in arm A and 5 (62.5%) in arm B received post-protocol therapy. EGFR tyrosine kinase inhibitors were most commonly used (12 patients in arm A and 10 in arm B). Pemtrexed and docetaxel tended to be more frequently used in arm A (10 and 15, respectively) than arm B (5 for each). Among 10 patients in arm B who dropped out protocol treatment during induction phase by the reason other than PD, 4 continued to receive gemcitabine plus carboplatin combination therapy as off-protocol treatment. No patient was treated with any maintenance therapy as post-protocol treatment.

## Discussion

Our study was launched on the assumption that gemcitabine monotherapy introduced immediately after standard induction platinum-doublet chemotherapy is a promising candidate for maintenance therapy for advanced NSCLC.

There was no significant difference in the transit rate to maintenance phase and treatment response during induction phase between the two arms of our study. Compared with the previous studies, 48% of patients in arm A received maintenance therapy, which was not inferior to the percentage (33.6%, 73/217) of those who transited to weekly paclitaxel continuation-maintenance from 4 cycles of carboplatin plus tri-weekly paclitaxel in a phase III study by Belani et al. [21]. On the other hand, only 33.0% of patients in arm B could receive maintenance therapy, which was lower than 54.8% (309/563) in a phase III study by Fidias et al. that had compared immediate versus delayed docetaxel monotherapy after 4 cycles of induction carboplatin plus gemcitabine [4]. As for treatment response to induction chemotherapy of carboplatin plus tri-weekly paclitaxel, the RR (36.0%) in arm A of our study was superior to that (19.2%) in the study by Belani et al. [21]. On the other hand, in comparison of the RR for carboplatin plus gemcitabine, 16.7% in arm B of our study was lower than 31.1% (168/563) in Fidias’s trial [4], but similar to 20.3% (13/64) in a randomized phase II study of West Japan Thoracic Oncology Group 0104 trial [22]. The most probable reason for these differences between the two arms of our study was that more patients were withdrawn from the protocol study due to AEs in arm B (n = 9) than in arm A (n = 1) and most of them (n = 7) were not assessable for the response evaluation in arm B, despite that there were no statistical differences in AEs between the two arms except that the peripheral sensory neuropathy was more frequent and severer in arm A. Furthermore, the certain cause of more frequent withdrawal in arm B was that discontinuation criteria were much stricter for arm B than arm A, because neither omission nor delay longer than 2 weeks of gemcitabine administration on day 8 was permitted.

The applicability of gemcitabine for maintenance use remains unestablished. There were 2 randomized phase III studies that had compared gemcitabine maintenance with best supportive care (BSC) in a continuation-maintenance setting after achieving objective response or disease stabilization by 4 cycles of initial gemcitabine plus platinum therapy for advanced NSCLC [6,7]. Both the studies showed that gemcitabine maintenance therapy significantly prolonged the time-to-progression (TTP) or PFS, but failed to improve the OS. In one study by Brodowicz et al., the median TTP from the time of enrollment was 6.6 months for gemcitabine and 5.0 months for BSC arms (*p* < 0.001), while median OS throughout study was 13.0 months for gemcitabine and 11.0 months for BSC arms (*p* = 0.195). Compared with this study, our study was inferior in PFS, but similar in OS. We could not compare our study with the other study by Perol et al. because neither PFS nor OS from the enrollment was reported. In addition, the median and average numbers of cycles of delivered gemcitabine in maintenance phase in our study were 4 and over 5.5, respectively, which were larger than median 3 cycles in the study by Brodowicz et al. [5] and similar to median 4 cycles in the study by Perol et al. [6]. Noteworthily, among 20 patients who received maintenance chemotherapy in our study, only 4 patients dropped out because they could not meet the criteria of the next course due to AEs. Thus, gemcitabine is characteristically well-tolerated and little cumulatively toxic, which enabled patients to continue the maintenance treatment for a longer time.

Pemetrexed [7] and bevacizumab [11] have been recently approved for non-squamous NSCLC as a maintenance therapy or a first-line treatment in combination with platinum-based chemotherapy. Maintenance therapy using these two drugs has been shown to bring about a significant survival benefit. In contrast, there are few effective drugs with enough evidences as maintenance therapy for NSCLC patients with squamous histology. Unlike pemetrexed and bevacizumab, gemcitabine has antitumor efficacy against squamous NSCLC. Therefore, a study evaluating gemcitabine as a maintenance therapy specific for squamous histology seems very interesting and promising.

## Conclusions

In conclusion, our study suggested that gemcitabine may be one of suitable drugs for maintenance therapy because of its well-tolerability and little cumulative toxicity. A study evaluating gemcitabine as a maintenance therapy is expected especially in patients with squamous NSCLC histology, for which few beneficial evidences have been demonstrated.

## Competing interests

The authors declare that they have no competing interests.

## Authors’ contributions

TK and KK conceived the study design. All authors contributed to patient recruitment, carried out clinical study procedures. SM and TK analyzed and interpreted the data, and prepared the manuscript. IT, KK and IK critically revised the manuscript for important intellectual content. All authors read and approved the final manuscript.
